# Trends and outcomes of late initiation of combination antiretroviral therapy driven by late presentation among HIV-positive Taiwanese patients in the era of treatment scale-up

**DOI:** 10.1371/journal.pone.0179870

**Published:** 2017-06-30

**Authors:** Kuan-Yin Lin, Chien-Yu Cheng, Chia-Wen Li, Chia-Jui Yang, Mao-Song Tsai, Chun-Eng Liu, Yuan-Ti Lee, Hung-Jen Tang, Ning-Chi Wang, Te-Yu Lin, Yi-Chien Lee, Shih-Ping Lin, Yu-Shan Huang, Jun-Yu Zhang, Wen-Chien Ko, Shu-Hsing Cheng, Chien-Ching Hung, for the Taiwan HIV Study Group

**Affiliations:** 1 Department of Medicine, National Taiwan University Hospital Jin-Shan Branch, New Taipei City, Taiwan; 2 Department of Internal Medicine, Taoyuan General Hospital, Ministry of Health and Welfare, Taoyuan City, Taiwan; 3 School of Public Health, National Yang-Ming University, Taipei, Taiwan; 4 Department of Internal Medicine, National Cheng Kung University Hospital, Tainan, Taiwan; 5 Department of Medicine, National Cheng Kung University Medical College, Tainan, Taiwan; 6 Department of Internal Medicine, Far Eastern Memorial Hospital, New Taipei City, Taiwan; 7 School of Medicine, National Yang-Ming University, Taipei, Taiwan; 8 Department of Internal Medicine, Changhua Christian Hospital, Changhua, Taiwan; 9 School of Medicine, Chung Shan Medical University, Taichung, Taiwan; 10 Department of Internal Medicine, Chung Shan Medical University Hospital, Taichung, Taiwan; 11 Department of Internal Medicine, Chi Mei Medical Center, Tainan, Taiwan; 12 Department of Health and Nutrition, Chia Nan University of Pharmacy and Sciences, Tainan, Taiwan; 13 Department of Internal Medicine, Tri-Service General Hospital and National Defense Medical Center, Taipei, Taiwan; 14 Department of Internal Medicine, Ditmanson Medical Foundation Chia-Yi Christian Hospital, Chia-Yi, Taiwan; 15 Department of Internal Medicine, Taichung Veterans General Hospital, Taichung, Taiwan; 16 Department of Internal Medicine, National Taiwan University Hospital Hsin-Chu Branch, Hsin-Chu, Taiwan; 17 Center of Infection Control, National Taiwan University Hospital, Taipei, Taiwan; 18 School of Public Health, Taipei Medical University, Taipei, Taiwan; 19 Department of Internal Medicine, National Taiwan University Hospital and National Taiwan University College of Medicine, Taipei, Taiwan; 20 Department of Parasitology, National Taiwan University College of Medicine, Taipei, Taiwan; 21 Department of Medical Research, China Medical University Hospital, Taichung, Taiwan; 22 China Medical University, Taichung, Taiwan; Azienda Ospedaliera Universitaria di Perugia, ITALY

## Abstract

**Objectives:**

The international and national HIV treatment guidelines in 2016 have focused on scaling up access to combination antiretroviral therapy (cART). We aimed to assess the trends and treatment outcomes of late cART initiation in Taiwan.

**Methods:**

Between June 2012 and May 2016, we retrospectively included antiretroviral-naive HIV-positive adults who initiated cART. Late initiation was defined as when cART was initiated in patients with a CD4 count <200 cells/mm^3^ or having experienced AIDS-defining illnesses. The treatment outcomes were assessed up to 6 months after starting cART.

**Results:**

We included 3655 HIV-positive patients, and the majority of the patients were male (95.4%) with a median age of 31 years and initiated non-nucleoside reverse-transcriptase inhibitor-containing regimens (87.0%). The median CD4 count at cART initiation increased from 207 cells/mm^3^ in 2012 to 298 cells/mm^3^ in 2016, and the overall proportion of late cART initiation decreased from 49.1% in 2012 to 29.0% in 2016 (*P* for trend <0.001). Late cART initiation mainly resulted from late presentation for HIV care and was associated with older age (per 1-year increase, adjusted odds ratio [AOR], 1.05; 95% CI, 1.04–1.06), HBsAg seropositivity (AOR, 1.31; 95% CI, 1.04–1.64), HIV care in central and southern Taiwan, initiating cART in earlier year, non-intravenous drug users (AOR, 1.96; 95% CI, 1.33–2.86), and negative hepatitis C serostatus (AOR, 1.47; 95% CI, 1.04–2.08). Compared with non-late initiators, late initiators had a higher rate of all-cause mortality (1.7% vs. 0.3%) and regimen modification due to virological failure (7.1% vs. 2.6%). The predicting factors of all-cause mortality were late cART initiation (adjusted hazard ratio [AHR], 5.40; 95% CI, 2.14–13.65) and older age (AHR, 1.06; 95% CI, 1.03–1.10).

**Conclusions:**

While the proportion of late cART initiation decreased over time in Taiwan, late initiation remained in a substantial proportion of HIV-positive patients. The late initiators had higher risk for poor outcomes. The need for strategies to earlier detection of HIV infection and expediting cART initiation should be highlighted, especially among the older population.

## Introduction

The scale-up combination antiretroviral therapy (cART) helps reduce AIDS-related deaths and new HIV infections, as well as decrease further expenses for medical services [1]. The global and national HIV treatment guidelines and programs support and facilitate the scale-up of cART. The US Department of Health and Human Services (DHHS) guidelines have recommended cART for all HIV-positive patients regardless of CD4 cell count since 2012 [2]. Further results from randomized trials also confirmed the benefits of immediate initiation of cART in all HIV-positive patients [3, 4]. The global target set by World Health Organization and the Joint United Nations Programme on HIV/AIDS (WHO/UNAIDS) in 2015 aimed to provide cART to 90% of all people with diagnosed HIV infection by expanding the use of cART to all HIV-positive patients [5]. At the end of 2015, 46% of people living with HIV worldwide were receiving cART [6].

Many countries in the Asia-Pacific region have made great strides in accessing cART through their HIV treatment guidelines and programs, and the region’s treatment coverage rate has increased from 19% in 2010 to 41% in 2015; however, the coverage rate still lagged behind the global coverage rate [6–8]. While studies conducted in Asia have demonstrated the increasing trends of CD4 cell count at cART initiation, the median CD4 cell count remained at around 200 cells/mm^3^ in 2011 [9]. The low median CD4 cell count at cART initiation suggests that late HIV diagnosis, delayed linkage to HIV care, and late cART initiation despite timely entry into care remain prevalent in this region [9].

These previous findings may not be generalized across all Asia-Pacific countries, however; whether the national HIV treatment programs get HIV-positive patients treated earlier may rely on regional studies. In Taiwan, a high-income country according to the World Bank classification [10], HIV-related medical services are provided free-of-charge. In response to expanding antiretroviral therapy through the national HIV treatment guidelines [11], assessing the trends and predictors of late cART initiation can improve engagement strategies in HIV care. In this study, we aimed to investigate the CD4 cell counts at cART initiation, to characterize the temporal trends of late cART initiation, and to evaluate the associated factors with late cART initiation and its impact on treatment outcomes in Taiwan.

## Patients and methods

### Study population and setting

We conducted a retrospective cohort study at 11 major designated hospitals for HIV care that participated in the Taiwan HIV Study Group. We included all HIV-positive patients aged 20 years or greater who were antiretroviral-naïve and initiated cART between 1 June 2012 and 31 May 2016. Patients without baseline CD4 cell count before cART initiation were excluded. Data was collected locally at each participating hospital, and then pooled and analyzed at the National Taiwan University Hospital. The study was approved by the Research Ethics Committee of National Taiwan University Hospital, Research Ethics Review Committee of Far Eastern Memorial Hospital, Medical Ethics and Institutional Review Board of Taoyuan General Hospital, and Institutional Review Boards (Tri-Service General Hospital, National Taiwan University Hospital Hsin-Chu Branch, Taichung Veterans General Hospital, Chung Shan Medical University Hospital, Changhua Christian Hospital, Chia-Yi Christian Hospital, National Cheng Kung University Hospital, and Chi Mei Medical Center). The informed consent was waived.

The Taiwan Centers for Disease Control (CDC) has provided HIV-positive patients with free-of-charge medical services, including cART, management of opportunistic illnesses, and laboratory testing, including monitoring of CD4 cell count and plasma HIV RNA load (PVL). Genotypic resistance assays were neither offered free-of-charge or routinely determined before cART initiation, although surveillance data suggested that the prevalence of transmitted drug resistance of HIV-1 to at least one antiretroviral was in the range of 10–15%; instead, the assays were performed at Taiwan CDC or a few designated hospitals for patients with virological failure and PVL >1000 copies/mL [12–14].The national HIV treatment guidelines had increased the CD4 cell count threshold for cART initiation from 350 to 500 cells/mm^3^ in September 2013 [11], which was further revised to treat all HIV-positive patients irrespective of CD4 cell count in June 2016. In light of increasing medical expenditure and budgetary constraints, Taiwan CDC has implemented regulations on the regimens of cART to be initiated in antiretroviral-naive patients since 1 June 2012. Between 1 June 2012 and 31 May 2016, antiretroviral-naive patients had been recommended to start cART with the preferred regimens of non-nucleoside reverse-transcriptase inhibitor (nNRTI)-containing regimens [15]. After 1 June 2016, the preferred regimens have been changed to 3 single-tablet regimens, including coformulated efavirenz/emtricitabine/tenofovir, rilpivirine/emtricitabine/tenofovir, and dolutegravir/abacavir/lamivudine. Rilpivirine and coformulated emtricitabine/tenofovir were not available in Taiwan until early 2014 and 2015, respectively. The study period was selected based on the date when major changes were made to the national HIV treatment guidelines.

### Data collection and definitions

We collected the information on demographics and clinical characteristics, such as age, sex, mode of HIV exposure, hepatitis B virus (HBV) surface antigen (HBsAg), hepatitis C virus (HCV) antibody, baseline CD4 cell count and PVL at cART initiation and serial follow-up visits, AIDS-defining illnesses before cART initiation, dates of cART initiation and loss to follow-up, and initial and switched antiretroviral regimens. The baseline CD4 cell count and PVL were defined as the data prior to and nearest to the date of cART initiation. Late initiation of cART was defined as when cART was initiated in patients with a baseline CD4 cell count <200 cells/mm^3^ or having experienced AIDS-defining illnesses before cART initiation [9, 16].

The treatment outcomes were assessed at 6 months and comparisons were made between HIV-positive patients with and without late cART initiation, and those initiating cART at CD4 cell count <500 versus ≧500 cells/mm^3^, or PVL <100,000 versus ≧100,000 copies/mL. Within 6 months after initiating cART, patients returned for assessment of virological, immunological, and clinical responses at week 4, and subsequently every 8 to 12 weeks [15]. The treatment outcomes assessed included all-cause mortality and regimen modification. Regimen modification, which was defined as the removal, addition or switch of at least one antiretroviral drug from the initial cART regimen within 6 months after cART initiation, and loss to follow-up. The reasons for modifying cART regimen were further categorized into 4 groups, including adverse event, treatment failure (e.g. virological failure, loss to follow-up, or cART interruption), simplification, and other reasons (e.g. drug-drug interaction, patient’s choice, or unknown cause). Virological failure was defined as a PVL >200 copies/mL at least 6 months after starting cART.

### Statistical analysis

Categorical variables were analyzed using the Chi-square test or Fisher’s exact test if the expected values were <10. Continuous variables were compared using the Wilcoxon-Mann-Whitney test. The trend analyses were evaluated by the generalized linear model and Cochran-Armitage trend test for continuous and categorical variables, respectively. The factors associated with late cART initiation were identified by logistic regression model. The predictors of all-cause mortality and regimen modification were determined by Kaplan-Meier survival estimations and Cox proportional hazards model. All variables in univariate analyses were selected for subsequent multivariable analyses. Ninety-five percent confidence intervals (CIs) of odds ratios (ORs) or hazard ratios (HRs) were computed to estimate the effects of each variable. All tests were two-tailed and *P* <0.05 was considered statistically significant. Statistical analyses were performed using Stata software version 12.0 (Stata Corporation, College Station, TX).

## Results

### Characteristics of study population

During the 4-year study period, 3655 HIV-positive Taiwanese patients initiating cART were included. The demographics and clinical characteristics of all included patients are summarized in **Table 1**. Most patients were male (95.4%) with a median age of 31 years and men who have sex with men (76.9%) and received HIV care at designated hospitals in northern Taiwan (70.9%). Approximately 10% and 20% of the patients were HBsAg-positive and anti-HCV-positive at cART initiation, respectively. The overall median baseline CD4 cell count was 270 cells/mm^3^ (interquartile range [IQR], 148–381 cells/mm^3^), and 13.2% of the patients had experienced AIDS-defining illnesses before cART initiation. Half (50.6%) of the patients initiated cART before the first half year of 2014, and approximately 87.0% started nNRTI-containing regimens.

**Table 1:** Patients’ characteristics at initiation of combination antiretroviral therapy (cART) stratified by late and non-late initiation of cART.

Characteristics	All patients (n = 3655)	Patients with late initiation (n = 1278)	Patients with non-late initiation (n = 2377)	P*
Age, median (IQR), years	31 (26–38)	33 (28–41)	30 (25–37)	<0.001
Sex, male, n (%)	3487 (95.4)	1219 (95.4)	2268 (95.4)	0.966
Mode of HIV exposure, n (%)				<0.001
Homosexual sex	2810 (76.9)	987 (77.2)	1823 (76.7)	
Heterosexual sex	220 (6.0)	109 (8.5)	111 (4.7)	
Intravenous drug use	591 (16.2)	162 (12.7)	429 (18.0)	
Others**	34 (0.9)	20 (1.6)	14 (0.6)	
Region of HIV care, n (%)***				<0.001
Northern Taiwan	2593 (70.9)	819 (31.6)	1774 (68.4)	
Central Taiwan	509 (13.9)	222 (43.6)	287 (56.4)	
Southern Taiwan	553 (15.1)	237 (42.9)	316 (57.1)	
HBsAg seropositivity, n (%)	390 (10.7)	172 (13.5)	218 (9.2)	<0.001
HCV seropositivity, n (%)	665 (18.2)	185 (14.5)	480 (20.2)	<0.001
Baseline CD4 cell count, median (IQR), cells/mm^3^	270 (148–381)	89 (34–156)	339 (272–442)	<0.001
Baseline PVL, median (IQR), log_10_ copies/mL	4.8 (4.3–5.2)	5.2 (4.8–5.6)	4.6 (4.2–5.0)	<0.001
AIDS-defining illness, n (%)	483 (13.2)	483 (37.8)	0 (0.0)	<0.001
Year of cART initiation, n (%)				<0.001
June 2012—May 2013	793 (21.7)	331 (25.9)	462 (19.4)	
June 2013—May 2014	1057 (28.9)	365 (28.6)	692 (29.1)	
June 2014—May 2015	1045 (28.6)	353 (27.6)	692 (29.1)	
June 2015—May 2016	760 (20.8)	229 (17.9)	531 (22.3)	
Type of cART, n (%)				0.640
NRTIs plus nNRTI	3180 (87.0)	1108 (86.7)	2072 (87.2)	
NRTIs plus PI	368 (10.1)	128 (10.0)	240 (10.1)	
NRTIs plus INSTI	107 (2.9)	42 (3.3)	65 (2.7)	

**Abbreviations:** CART, combination antiretroviral therapy; HBsAg, hepatitis B surface antigen; HCV, hepatitis C virus; INSTI, integrase strand transfer inhibitor; IQR, interquartile range; NRTI, nucleoside reverse-transcriptase inhibitor; nNRTI, non-nucleoside reverse-transcriptase inhibitor; PI, protease inhibitor; PVL, plasma HIV RNA load.

*The statistical significance was tested for the differences between patients with late and non-late cART initiation.

**Others included patients with exposure to blood products and unknown exposures.

***Five hospitals located in northern Taiwan (National Taiwan University Hospital, Tri-Service General Hospital, Far Eastern Memorial Hospital, Taoyuan General Hospital, and National Taiwan University Hospital Hsin-Chu Branch); 3 hospitals in central Taiwan (Taichung Veterans General Hospital, Chung Shan Medical University Hospital, and Changhua Christian Hospital) and 3 hospitals in southern Taiwan (Chia-Yi Christian Hospital, National Cheng Kung University Hospital, and Chi Mei Medical Center).

Among those 3655 included patients, the dates of and CD4 cell counts at HIV diagnosis were available in 953 patients (26.1%), in whom 340 (35.7%) presented for HIV care with CD4 cell counts <200 cells/mm^3^. The median duration between HIV diagnosis and cART initiation was 1.6 months (range, 0–182.3 months) in these patients. The interval between HIV diagnosis and cART initiation according to the year of HIV diagnosis decreased from 3.5 months in 2012 to 0.5 months in 2016.

### Trends of CD4 cell count at cART initiation

**Fig 1:**
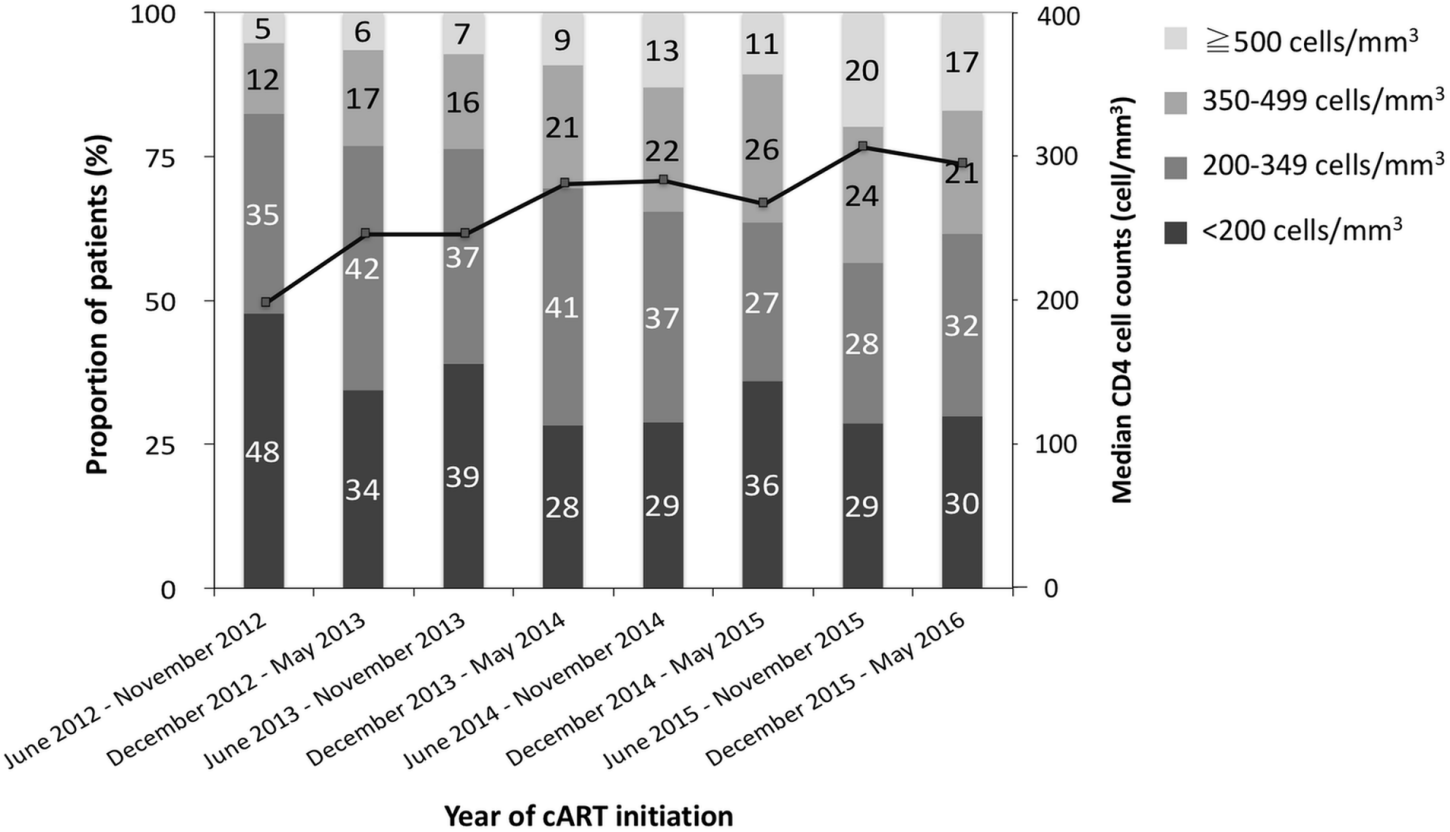
Trends in median and distribution of CD4 cell counts at initiation of combination antiretroviral therapy (cART) from 1 June 2012 to 31 May 2016.

The trends of median CD4 cell counts at cART initiation that were assessed over each 6-month study period are shown in **Fig 1**. The median CD4 cell count significantly increased from 207 cells/mm^3^ for the patients initiating cART in the second half year of 2012 to 298 cells/mm^3^ for those in the first half year of 2016 (*P* for trend <0.001). The CD4 cell counts were classified into 4 groups: <200 cells/mm^3^, 200–349 cells/mm^3^, 350–499 cells/mm^3^, and ≧500 cells/mm^3^. In the second half year of 2012, the majority of the patients (47.7%) initiated cART with a CD4 cell count <200 cells/mm^3^; in contrast, more than one-third of the patients (38.4%) started cART with a CD4 cell count ≧350 cells/mm^3^ in the first half year of 2016. While the proportions of baseline CD4 cell counts between 350–499 cells/mm^3^ and ≧500 cells/mm^3^ from 2013 onwards were significantly higher than those in 2012, the proportions of CD4 cell counts between 200–349 cells/mm^3^ and <200 cells/mm^3^ decreased over time (*P* for trend <0.001). However, there were still 30% of the patients who initiated cART late with CD4 cell counts <200 cells/mm^3^ in the most recent study year. In addition, the median CD4 cell counts had no significant change among patients aged 50 years or greater, among whom the median CD4 cell count was 130 cells/mm^3^ in the second half year of 2012 and 165 cells/mm^3^ in the first half year of 2016.

### Trends and associated factors with late cART initiation

**Fig 2:**
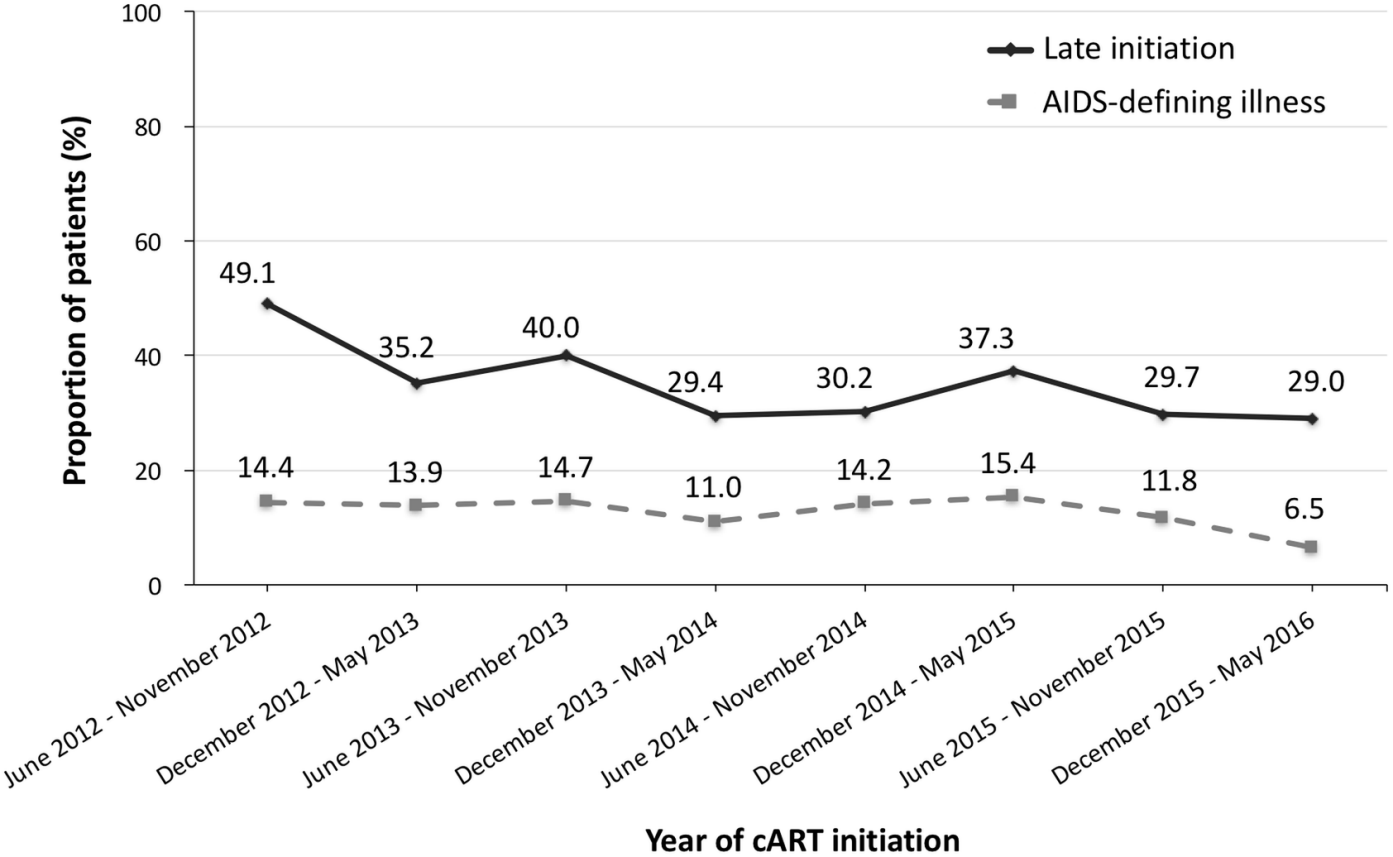
Changes over time in proportion of patients with late initiation of combination antiretroviral therapy (cART) and AIDS-defining illnesses from 1 June 2012 to 31 May 2016.

Among the 3655 patients, 1278 (35.0%) initiated cART late and 2377 (65.0%) did not. In **Fig 2**, the proportion of the patients with late cART initiation had significantly declined from 49.1% in the second half year of 2012 to 29.0% in the first half year of 2016 (*P* for trend <0.001). However, the percentage of the patients having AIDS-defining illnesses prior to cART initiation remained at more than 10% till the first half year of 2016, without statistically significant difference compared with those of previous study years (*P* for trend = 0.082).

The demographics and clinical characteristics of the patients stratified by late and non-late cART initiation are shown in **Table 1**. The patients with late cART initiation were older and more likely to be heterosexual, seropositive for HBsAg, and seronegative for HCV antibody, and to receive HIV care at hospitals in central and southern Taiwan. They also tended to have lower baseline CD4 cell counts and higher PVL, and started cART in the earlier years (all *P* <0.05). In multivariable analysis, the factors associated with late cART initiation were older age (per 1-year increase, adjusted OR [AOR], 1.05; 95% CI, 1.04–1.06), receiving HIV care at hospitals in central and southern Taiwan (AOR, 1.78; 95% CI, 1.45–2.19 and 1.65; 95% CI, 1.35–2.00, respectively), and HBsAg seropositivity (AOR, 1.31; 95% CI, 1.04–1.64). Late cART initiators were more likely to be non-intravenous drug users (IDUs) (AOR, 1.96; 95% CI, 1.33–2.86) and to test negative for anti-HCV (AOR, 1.47; 95% CI, 1.04–2.08). In line with the aforementioned trend analysis for baseline CD4 cell count, there was a statistically significant association between non-late cART initiation and initiating cART in later years, which strengthened over time during the study period (**Table 2**).

**Table 2:** Logistic analysis to identify the factors associated with late initiation of combination antiretroviral therapy.

Variables	Univariate analysis		Multivariable analysis*	
	OR (95% CI)	*P*	AOR (95% CI)	*P*
Age, per 1-year increase	1.04 (1.03–1.04)	<0.001	1.05 (1.04–1.06)	<0.001
Male sex	0.99 (0.72–1.37)	0.966	0.95 (0.64–1.40)	0.784
Mode of HIV exposure				
Homosexual sex	1.00 (reference)		1.00 (reference)	
Heterosexual sex	1.81 (1.38–2.39)	<0.001	0.99 (0.71–1.39)	0.970
Intravenous drug use	0.70 (0.57–0.85)	<0.001	0.51 (0.35–0.75)	0.001
Region of HIV care				
Northern Taiwan	1.00 (reference)		1.00 (reference)	
Central Taiwan	1.68 (1.38–2.03)	<0.001	1.78 (1.45–2.19)	<0.001
Southern Taiwan	1.62 (1.35–1.96)	<0.001	1.65 (1.35–2.00)	<0.001
HBsAg seropositivity	1.54 (1.25–1.91)	<0.001	1.31 (1.04–1.64)	0.020
HCV seropositivity	0.67 (0.56–0.81)	<0.001	0.68 (0.48–0.96)	0.030
Year of cART initiation				
June 2012—May 2013	1.00 (reference)		1.00 (reference)	
June 2013—May 2014	0.74 (0.61–0.89)	0.002	0.77 (0.63–0.93)	0.008
June 2014—May 2015	0.71 (0.59–0.86)	<0.001	0.72 (0.59–0.88)	0.001
June 2015—May 2016	0.60 (0.49–0.74)	<0.001	0.65 (0.52–0.81)	<0.001

**Abbreviations:** AOR, adjusted odds ratio; CI, confidence interval; HBsAg, hepatitis B surface antigen; HCV, hepatitis C virus; OR, odds ratio.

*All variables in univariate analyses were selected for subsequent multivariable analyses.

### Treatment outcomes and predicting factors

**Fig 3:**
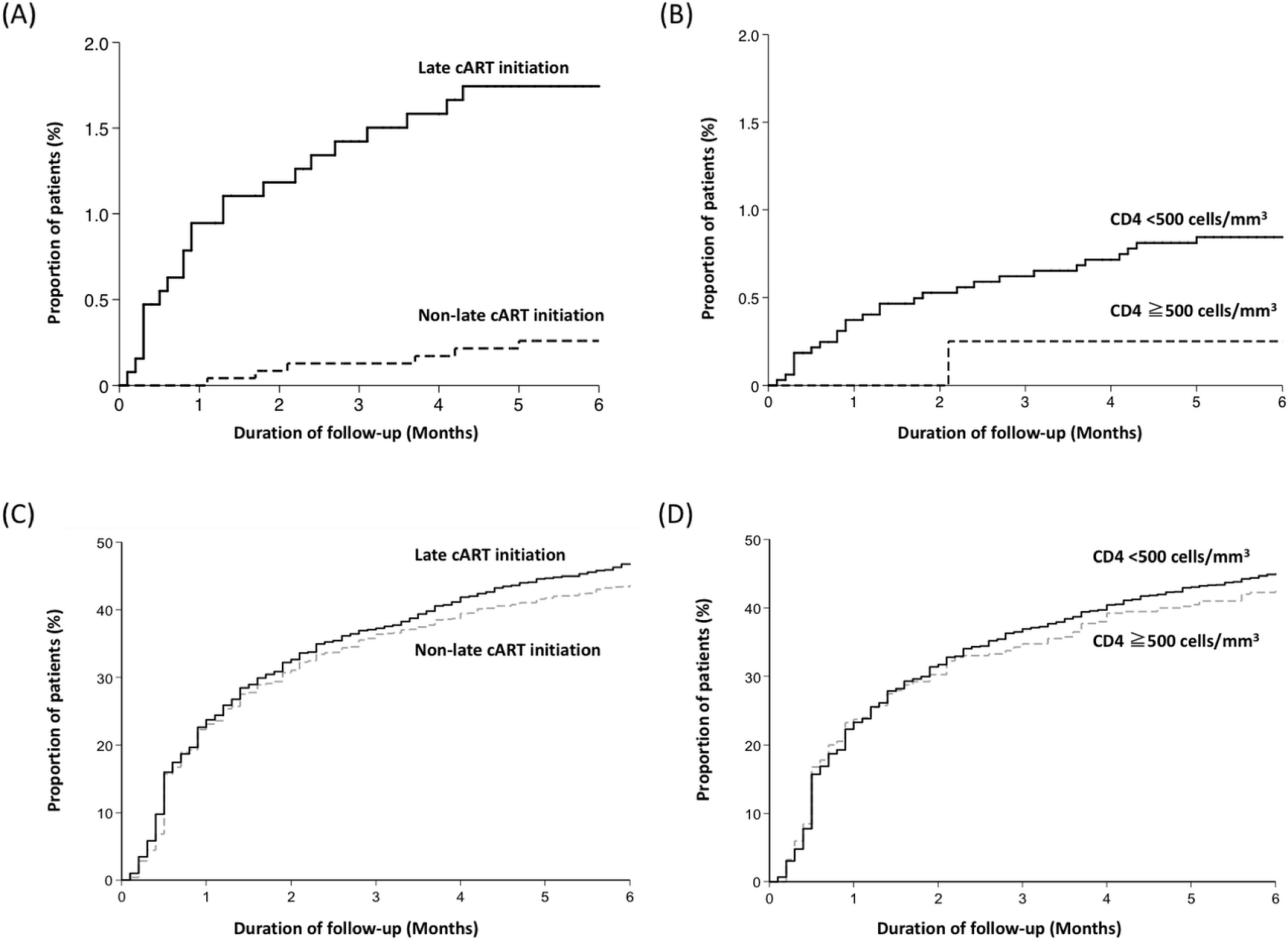
Kaplan-Meier estimates of the cumulative proportion of patients with treatment outcomes. All-cause mortality stratified by late initiation and non-late initiation (A), All-cause mortality stratified by baseline CD4 cell counts <500 and ≧500 cells/mm^3^ (B), Regimen modification stratified by late initiation and non-late initiation (C), and Regimen modification stratified by baseline CD4 cell counts <500 and ≧500 cells/mm^3^ (D).

The comparisons of treatment outcomes between late and non-late initiation are shown in **Table 3**, while the comparisons between patients initiating cART at CD4 cell counts <500 versus ≧500 cells/mm^3^ are shown in **S1 Table**. All-cause mortality occurred in 22 (1.7%) and 6 (0.3%) patients in the late initiation and non-late initiation groups, respectively. The estimated HR for all-cause mortality in the late initiation group was 6.86 (95% CI, 2.78–16.91) compared with the non-late initiation group in univariate analysis (**Fig 3A**). However, the difference in all-cause mortality between the patients initiating cART at CD4 cell counts <500 and those at CD4 cell counts ≧500 cells/mm^3^ did not reach statistical significance (HR, 3.37; 95% CI, 0.46–24.78) (**Fig 3B**). In multivariable analysis, late cART initiation remained statistically significantly associated with all-cause mortality. The other independent predictor of all-cause mortality was older age (per 1-year increase, adjusted HR [AHR], 1.06; 95% CI, 1.03–1.10) (**Table 4**).

**Table 3:** Comparisons of treatment outcomes in patients with late and non-late combination antiretroviral therapy initiation.

Outcomes	Patients with late initiation (n = 1278)	Patients with non-late initiation (n = 2377)	HR (95% CI)	*P*
All-cause mortality, n (%)	22 (1.7)	6 (0.3)	6.86 (2.78–16.91)	<0.001
Regimen modification, n (%)*	598 (46.8)	1041 (43.8)	1.10 (0.99–1.21)	0.077
Adverse event	422 (33.0)	834 (35.1)	0.94 (0.83–1.05)	0.264
Treatment failure**	120 (9.4)	120 (5.1)	2.02 (1.56–2.61)	<0.001
Virological failure	91 (7.1)	62 (2.6)	2.82 (2.04–3.90)	<0.001
Baseline PVL ≥100,000	69 (5.4)	24 (1.0)	2.04 (1.28–3.25)	0.003
copies/mL				
Baseline PVL <100,000	22 (1.7)	38 (1.6)	2.27 (1.34–3.84)	0.002
copies/mL				
Loss to follow-up or	29 (2.3)	58 (2.4)	1.03 (0.64–1.64)	0.911
interruption				
Simplification	47 (3.7)	70 (2.9)	1.23 (0.85–1.78)	0.281
Others	14 (1.1)	20 (0.8)	1.21 (0.60–2.43)	0.591

**Abbreviations:** CI, confidence interval; HR, hazard ratio; PVL, plasma HIV RNA load.

*Regimen modification included the removal, addition, and switch of at least one antiretroviral drug from the initial cART regimen, and loss to follow-up within 6 months after starting cART.

**The causes of treatment failure included virological failure, loss to follow-up, and cART interruption. Virological failure was defined as a PVL >200 copies/mL at least 6 months after starting cART.

**Table 4:** Cox-regression hazards model for factors predicting all-cause mortality.

Variables	Univariate analysis		Multivariable analysis*	
	HR (95% CI)	*P*	AHR (95% CI)	*P*
Late initiation	6.86 (2.78–16.91)	<0.001	5.40 (2.14–13.65)	<0.001
Age, per 1-year increase	1.07 (1.05–1.10)	<0.001	1.06 (1.03–1.10)	<0.001
Male sex	1.31 (0.18–9.64)	0.791	1.97 (0.26–14.82)	0.508
HBsAg seropositivity	1.39 (0.48–4.02)	0.539	0.84 (0.29–2.43)	0.742
HCV seropositivity	1.25 (0.51–3.07)	0.633	0.96 (0.38–2.43)	0.933
Year of cART initiation				
June 2012—May 2013	1.00 (reference)		1.00 (reference)	
June 2013 –May 2014	1.89 (0.59–6.02)	0.283	2.37 (0.74–7.57)	0.147
June 2014 –May 2015	1.93 (0.60–6.15)	0.267	2.42 (0.75–7.75)	0.138
June 2015 –May 2016	1.04 (0.26–4.17)	0.953	1.37 (0.34–5.51)	0.654

**Abbreviations:** AHR, adjusted hazard ratio; cART, antiretroviral therapy; CI, confidence interval; HBsAg, hepatitis B surface antigen; HCV, hepatitis C virus; HR, hazard ratio.

*All variables in univariate analyses were selected for subsequent multivariable analyses.

Within 6 months after cART initiation, more than 40% of the patients had to modify their first antiretroviral regimens; the major reason for modification was cART-associated adverse events, followed by treatment failure and simplification (**Table 3**). The percentages of overall regimen modification, regimen modification due to adverse events, and simplification were all similar when the comparisons were stratified by late cART initiation (**Fig 3C**) or initiating cART at CD4 cell counts <500 cells/mm^3^ (**Fig 3D**). Patients initiating cART late and at CD4 cell counts <500 cells/mm^3^ were more likely to modify cART for virological failure, with the estimated HR of 2.82 (95% CI, 2.04–3.90) and 2.58 (95% CI, 1.21–5.52) in univariate analysis, respectively. The negative impact of late cART initiation on virological response was observed not only in the patients initiating cART at PVL <100,000 but also those at PVL ≧100,000 copies/mL (Table 3).

## Discussion

Despite the substantial number of studies on the trends of CD4 cell count at cART initiation before 2013, this study aimed to evaluate the temporal changes and outcomes of late cART initiation during the period of rapid treatment scale-up based on recent guidelines [17–19]. Our study demonstrates declining proportions of late initiation over the 4-year study period. Initiating cART late was mainly driven by late presentation for care, which had significant impact on all-cause mortality and regimen modification due to virological failure.

The trends of CD4 cell count at cART initiation provide valuable insight into how well HIV programs are implemented in response to HIV epidemic [20]. Since initiating cART at a higher CD4 cell count prevents HIV-associated illnesses, averts new infections, and saves money to achieve the target of ending the AIDS epidemic [5], current HIV treatment guidelines have evolved to recommend initiating cART regardless of CD4 cell counts [2, 21]. Both the WHO and Taiwanese HIV treatment guidelines had increased the CD4 threshold from 350 cells/mm^3^ to 500 cells/mm^3^ in 2013 [11, 19]. In our study, the overall median baseline CD4 cell count remained below 300 cells/mm^3^ and less than half of the patients started cART at a CD4 cell count >350 cells/mm^3^. In addition, the proportion of late cART initiators was substantial, with 29% in the first half year of 2016. Nearly half of late cART initiators had prior AIDS-defining illnesses, for which no statistically significant temporal declines were found. The percentage of late cART initiation remained high because it was the consequence of late presentation for HIV care. The findings of our study and the previous studies all suggest that HIV-positive patients still accessed HIV care and treatment late worldwide. According to the meta-regression from Western developed countries, the mean CD4 cell count at presentation for HIV care increased minimally by 1.5 cells/mm^3^ per year, from 307 cells/mm^3^ in 1992 to 336 cells/mm^3^ in 2011 [17]. A large multi-national cohort study in Asia, which recruited patients in all categories of country income, reported temporal trends similar to our study [9], in which the median CD4 cell count at cART initiation increased from 115 cells/mm^3^ in 2008 to 302 cells/mm^3^ after 2011, and proportion of late cART initiation decreased from 81.7% to 36.3%.

The factors associated with late cART initiation identified in our study were partly consistent with previous studies, which reported that male sex, older age, earlier year of cART initiation, heterosexual sex as the HIV exposure category, marital status, and higher education level were associated with a greater likelihood of late cART initiation [9, 22, 23]. The older HIV-positive patients, especially those aged 50 years or greater, had lower CD4 cell counts at cART initiation without statistically significant trends compared with all included HIV-positive patients. Entry into HIV care at an advanced age is not only associated with higher mortality in both the natural and treated history of HIV infection but with adverse consequences of delayed cART initiation [24, 25]. The contributing factors to the findings that older HIV-positive patients were diagnosed and treated at a more advanced stage may include limited sexual health information targeting older adults, poor awareness of the risk of HIV infection, and failure of physicians to consider the possibility of HIV infection [26, 27]. The Taiwanese national surveillance data also reported the similar epidemiological picture; the percentages of newly diagnosed HIV infection and AIDS in patients between the age of 40 and 64 years were 15.3% and 28.7%, respectively [28].

The differences in the timing of cART initiation across geographical regions may reflect diverse socioeconomic status of the patients and the clinical practices of physicians. People living in central and southern Taiwan have lower accessibility of medical resources and educational status, which may hamper scale-up of HIV treatment [23, 29, 30]. The finding of lower baseline CD4 cell count among HIV/HBV co-infected patients when cART was initiated may be related to the adverse impact of HBV on immunologic progression, especially in patients with positive hepatitis B e-antigen and a high HBV DNA load [31, 32]. Therefore, the recommendation of immediate cART initiation in HBV co-infected patients should be reinforced [2]. In contrast, HIV/HCV co-infection was inversely related with late cART initiation. The previous outbreak of HIV infection among Taiwanese IDUs had led to the high prevalence of HIV/HCV co-infection among the IDUs in our study (82.3%) [33]. Given that fact that HIV screening is mandatory for inmates and persons on entry into correctional facilities, who also receive free-of-charge HIV care under the national HIV treatment program in Taiwan, it is not surprising that linkage to HIV care and cART initiation during the incarceration periods is much easier for IDUs with HCV infection than other risk groups [33].

The large Asian observational cohort with the similar mortality rate also reported that late cART initiation was associated with mortality [9]. The meta-analysis of clinical trials has demonstrated that late cART initiation was consistently associated with poorer CD4 cell count recovery, which was not modulated by the individual cART regimen [34]. In our study, we did not observe the impact of late cART initiation on reasons of regimen modification other than due to virological failure. The absence of difference in the overall percentage of regimen modification between patients with and those without late cART initiation was also observed in an Australian cohort study [35]. The relationship between initiating cART at a CD4 cell count <500 cells/mm^3^ and regimen modification due to virological failure also supported the recommendation of universal access to cART [2, 21].

Our study has several limitations. First, the lack of information on other patient-level characteristics, such as education, marital status and socioeconomic status, may preclude us from identifying the association between those patient-level characteristics with late cART initiation. The association between seeking HIV care in different regions in Taiwan and late cART initiation probably reflects the impact of patient-level characteristics. Second, though almost every Taiwanese is covered by the National Health Insurance and HIV care is free of charge in Taiwan, the extent to which the late cART initiation in our study was driven by late HIV diagnosis, delayed linkage into HIV care, and late cART initiation was unclear. The substantial proportion of patients presenting for HIV care with CD4 cell counts less than 200 cells/mm^3^ suggested late diagnosis may play an important role in late cART initiation. Finally, genotypic resistance assays were not routinely determined in Taiwanese treatment-naïve HIV-positive patients and thus the impact of transmitted drug resistance of HIV-1 on the short-term treatment outcomes was unclear.

In conclusion, the median CD4 cell count at cART initiation increased and the proportion of late initiation decreased over time among HIV-positive Taiwanese patients in the era of treatment scale-up. However, the median CD4 cell count remained lower than the recommended threshold of cART initiation and the percentage of late initiators was still substantial. The late initiators had increased probability of all-cause mortality and regimen modification due to virological failure. The strategies to facilitating earlier diagnosis of HIV infection and access to cART are urgently needed, especially among the older population.

## Supporting information

S1 Table Compared treatment outcomes in patients with combination antiretroviral therapy initiation at CD4 cell counts ≧500 cells/mm3 and <500 cells/mm3. (DOCX)

S1 Data The minimal data set of the patients in this study. (XLSX)

## Abbreviations

cART: combination antiretroviral therapy
