# Supplementary material for: Trends and outcomes of late initiation of combination antiretroviral therapy driven by late presentation among HIV-positive Taiwanese patients in the era of treatment scale-up
Source: PLoS One. 2017 Jun 30;12(6):e0179870. doi: 10.1371/journal.pone.0179870 (PMC5493332; doi:10.1371/journal.pone.0179870)
Supplement: S1 Table — (DOCX) [file pone.0179870.s001.docx]

**Supplementary Table**

**S1 Table. Compared treatment outcomes in patients with combination antiretroviral therapy initiation at CD4 cell counts ≧500 cells/mm^3^ and <500 cells/mm^3^.**

| **Outcomes** | **CD4 cell counts** <**500 cells/mm^3^ (n=3254)** | **CD4 cell counts ≧500 cells/mm^3^ (n=401)** | **HR (95% CI)** | ***P*** |
| --- | --- | --- | --- | --- |
| All-cause mortality, n (%) | 27 (0.8) | 1 (0.2) | 3.37 (0.46-24.78) | 0.233 |
| Regimen modification, n (%)* | 1468 (45.1) | 171 (42.6) | 1.06 (0.91-1.25) | 0.438 |
| Adverse event | 1119 (34.4) | 137 (34.2) | 0.99 (0.83-1.19) | 0.946 |
| Treatment failure** | 225 (6.9) | 15 (3.7) | 1.94 (1.13-3.34) | 0.016 |
| Virological failure | 146 (4.5) | 7 (1.8) | 2.58 (1.21-5.52) | 0.013 |
| Baseline PVL ≧100,000  copies/mL | 92 (2.8) | 1 (0.2) | 3.58 (0.50-25.69) | 0.205 |
| Baseline PVL <100,000  copies/mL | 54 (1.7) | 6 (1.5) | 1.61 (0.69-3.74) | 0.269 |
| Loss to follow-up or  interruption | 79 (2.4) | 8 (2.0) | 1.28 (0.59-2.77) | 0.538 |
| Simplification | 101 (3.1) | 16 (4.0) | 0.77 (0.45-1.30) | 0.327 |
| Other reasons | 29 (0.9) | 5 (1.2) | 0.69 (0.27-1.78) | 0.442 |
| **Abbreviations:** CI, confidence interval; HR, hazard ratio; PVL, plasma HIV RNA load.  *Regimen modification included the removal, addition, and switch of at least one antiretroviral drug from the initial cART regimen, and loss to follow-up within 6 months after starting cART.  **The causes of treatment failure included virological failure, loss to follow-up, and cART interruption. Virological failure was defined as a PVL >200 copies/mL at least 6 months after starting cART. | | | | |
